# Struggling to fit the white coat and the role of contextual factors within a hospital organisation - an ethnographic study on the first months as newly graduated doctors

**DOI:** 10.1186/s12909-021-02493-2

**Published:** 2021-01-25

**Authors:** Tine Lass Klitgaard, Diana Stentoft, Mads Skipper, Mette Grønkjær, Susanne Backman Nøhr

**Affiliations:** 1 Department of Postgraduate Medical Education, Aalborg University Hospital, Aalborg, Denmark; 2 Department of Clinical Medicine, Aalborg University, Aalborg, Denmark; 3 Centre for Health Science Education and Problem-Based Learning, Aalborg University, Aalborg, Denmark; 4 Postgraduate Medical Educational Region North, Viborg, Denmark; 5 Department of Clinical Medicine, Aarhus University, Aarhus, Denmark; 6 Clinical Nursing Research Unit, Aalborg University Hospital, Aalborg, Denmark

**Keywords:** Contextual factors, Cultural historical activity theory (CHAT), Ethnography, Newly graduated doctors, Medical education, Postgraduate, Qualitative research, Struggles, Transition, Workplace organisation

## Abstract

**Background:**

Despite increased focus on improving the transition from being a medical student to working as a junior doctor, many newly graduated doctors (NGD) report the process of fitting the white coat as stressful, and burnout levels indicate that they might face bigger challenges than they can handle. During this period, the NGDs are in a process of learning how to be doctors, and this takes place in an organisation where the workflow and different priorities set the scene. However, little is known about how the hospital organisation influences this process. Thus, we aimed to explore how the NGDs experience their first months of work in order to understand 1) which struggles they are facing, and 2) which contextual factors within the hospital organisation that might be essential in this transition.

**Methods:**

An ethnographic study was conducted at a university hospital in Denmark including 135 h of participant observations of the NGDs (*n* = 11). Six semi-structured interviews (four group interviews and two individual interviews) were conducted (*n* = 21). The analysis was divided into two steps: Firstly, we carried out a “close-to-data” analysis with focus on the struggles faced by the NGDs. Secondly, we reviewed the struggles by using the theoretical lens of Cultural Historical Activity Theory (CHAT) to help us explore, which contextual factors within the hospital organisation that seem to have an impact on the NGDs’ experiences.

**Results:**

The NGDs’ struggles fall into four themes: *Responsibility*, *local knowhow, time management and collaborators.* By using the CHAT lens, we were able to identify significant contextual factors, including a physically remote placement, a missing overlap between new and experienced NGDs, a time limited introduction period, and the affiliation to several departments. These struggles and factors were highly intertwined and influenced by one another.

**Conclusion:**

Contextual factors within the hospital organisation may aggravate the struggles experienced by the NGDs, and this study points to possible elements that could be addressed to make the transition less challenging and overwhelming.

## Background

The development of undergraduate and postgraduate medical education has been devoted much attention in an attempt to diminish the gap between medical school and the work as doctors. Newly graduated doctors (NGD) enter a complex and busy environment where they are expected to contribute to the workforce within the first few weeks [1, 2]. In this transition, they become acquainted with the challenges of workplace learning: Although, the learning process of fitting the white coat is recognised as a legitimate purpose, it is to take place in a context where the workflow and the priority of different collaborators set the scene. This means that there often is neither the time nor the priorities to support the NGDs in this process [3]. Several studies report that the transition period is associated with both positive and negative experiences. Although the transition can be seen as an important learning experience with increasing responsibilities and tasks [4–7], many NGDs find it stressful and challenging [5, 7–12]. Burnout levels indicate that they may be facing bigger challenges than they can handle [1, 13].

Various factors have been identified as contributing to the NGDs’ feelings of stress and burnout. Several studies point to extensive working hours, sleep deprivation, challenges in clinical decision-making and high levels of responsibility as essential factors [7, 8, 14–17]. A Danish investigation among all residents in 2012 demonstrated that the doctors experienced a high level of time pressure and heavy workload during evening and night shifts. The perceived pressure was highest among recently graduated doctors as 69% of graduates stated that there was a “high” or “very high” level of time pressure during night shifts [18]. Lastly, international and national studies state that the gap should be elucidated as a clash between the ideals taught at medical school and the realities of clinical practice [1, 11, 19–22]. Thus, the transition from medical school to clinical practice represents a difficult and uncertain period to the NGDs.

Lefroy et al. [22] state that a lack of contextual knowledge, such as how to gain access to appropriate support, could affect the new doctors’ experiences of failure and may result in inadequate solutions. Moreover, Kilminster et al. [23] advocate that the doctors’ practice is mainly dependent on situational and contextual factors, rather than on formal frameworks. However, little is known about which contextual factors within the hospital organisation that might influence the doctors’ experiences. Inspired by Coles et al. [24], context is not perceived as the backdrop to the NGDs’ work. Rather, context can be widely perceived as elements that interact, influence, modify, facilitate or constrain the experiences of working as an NGD. Thus, there is a need to explore contextual factors in-depth in order to point at areas Within the organisation of medical education at hospitals, which could be optimised in order to make the transition less challenging.

The aim of this study is to explore how the NGDs experience their first months of work in a complex clinical setting in order to understand 1) which struggles they are facing, and 2) which contextual factors within the hospital organisation that might be essential in this transition. In this exploration, we include two different, yet interrelated, analytical strategies. In the first, we explore the struggles experienced by the NGDs. In the second, we add the hospital organisation as the unit of analysis and through the theoretical lens of Cultural Historical Activity Theory (CHAT), we explore which contextual factors within the hospital organisation that might have an impact on the NGDs’ experience of struggles when fitting in the white coat. This combination provides significant insights into the lived world of the NGDs and how contextual factors of the surrounding hospital organisation influence these experiences.

## Methods

### Methodology

To explore the complexity of the NGDs’ work and the struggles they are facing, an ethnographic study design was chosen employing the methods of participant observation and interviews. These methods gave us the opportunity to explore not only how the doctors themselves experience their work retrospectively (interviews), but also their practices and the surroundings *in* the situation (observations).

### Setting and participants

**Fig. 1:**
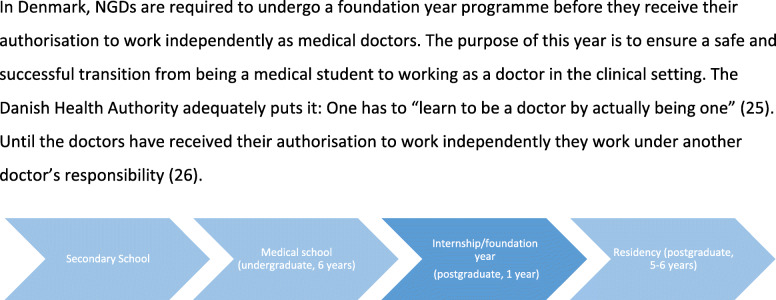
Medical Education in Denmark

The study took place at a university hospital in Denmark, where approximately 70 NGDs are employed yearly. The doctors included in this study were in the first part of their internship/foundation year programme (see further information about medical education in Denmark in Fig. 1, [25, 26]). Although the first year is referred to as training and is part of an educational programme, it is also a fulltime job (37 h/week in average), where the NGDs are expected to contribute to the workforce already within the first few weeks.

The fieldwork was conducted in the Accident and Emergency Department (A&E) and the cooperating medical departments. The A&E serves as the entry point of (nearly) all acutely admitted patients. NGDs from the A&E and the medical departments share the task of attending to the (medical) patients and deciding who are discharged and who are admitted for further diagnosing and treatment. In this complex context, the NGDs have to acquire specific competencies simultaneously, and they are assessed as part of their training programme during their employment [27, 28].

All involved departments were informed about the study and accepted to participate. Still, access also had to be planned with the NGDs as their participation depended on their consent [29]. NGDs were asked to sign a consent form indicating their agreement to participate and for their data to be used. The participants were chosen on the basis of availability (residents on duty on observations days) and with a variety in gender, medical school, department of employment and prior clinical experience in mind [30].

### Data generation – methods of the fieldwork

#### Participant observation and interviews

The first author is an anthropologist and conducted the fieldwork, where a total of 135 h of participant observation were carried out from June 2016 to March 2017. Even though the focus was on the NGDs, it quickly became clear that the NGDs constantly interacted with other staff (doctors and nurses) in their work, and thus these collaborators unavoidably became a part of the observations as well.

Participant observation involved participating in the doctors’ work life over a period of time, asking questions, listening and taking notes [31]. The objective was to explore the experiences of working as a NGD and to gain a better understanding of the complexity and multitude of factors involved in their work [30]. A total of 11 NGDs were observed throughout their working hours at different shifts and at different times of the day and the week. The first author donned the white coat and followed the NGDs throughout their entire shift observing patient examinations, staff meetings, administrative work, phone calls, coffee breaks etc. During the fieldwork, extensive field notes were written, including both observational notes of activities and interactions and more reflective notes of analytical ideas and the researcher’s position in the field. First as jotted notes and later into full, elaborated notes [30]. Whenever the NGDs interacted with patients or colleagues, the fieldworker remained in the background, but was occasionally asked to participate in the work, e.g. when assisting a patient while the NGD would auscultate the patient’s lungs.

The participant observations were supplemented by semi-structured interviews to gain insight into the NGDs’ subjective perception of their work and to be able to generate more specific interview questions [32]. The interviews took place at the hospital during the NGDs’ working hours, lasted between 1 and 2 h, and were audio-recorded. We chose to include group interviews to allow the NGDs to discuss different themes in order to see various or even contrasting perspectives on their first months of practice. (*N* = 6, NGDs = 21). However, due to practical reasons, individual interviews were conducted as well (*N* = 2) [30, 33, 34].

### Data analysis

In order to examine both the struggles experienced by the NGDs as well as the contextual factors influencing these experiences, we designed a two-step analysis. Firstly, we carried out a “close-to-data” analysis with focus on the NGDs’ experiences and the struggles they were facing. Secondly, we reviewed the findings with the theoretical lens of Cultural Historical Activity Theory (CHAT) to explore which contextual factors within the hospital organisation that might have an impact on the NGDs’ experience of struggles when the object of the activity is to fit the white coat.

#### First round of analysis – close-to-data

All interview recordings were transcribed verbatim. All field notes and interview transcriptions were analysed using NVivo (qualitative data analysis software). The first part of the analysis was inspired by Clarke and Braun’s thematic analysis [35]. The ethnographic research is an iterative-inductive process, and it can be difficult to separate the different phases, including the analytical one. According to this, the analysis already began during the fieldwork where the first author read and reread the field notes and transcripts to familiarise herself with the data. This included coding and searching for themes. The first and the last author performed the preliminary coding of data. All authors discussed the findings during the process of analysis. The codes were then clustered into themes by identifying patterns and similarities.

#### Second round of analysis – theory-guided

**Fig. 2:**
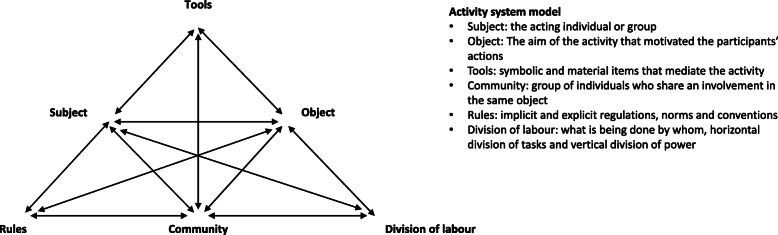
Activity system model, adapted from Engeström [37]

To explore the complexity and to point at contextual factors within the hospital organisation that might have an impact on the NGDs’ experience of struggles, we reviewed the themes again, this time using Cultural Historical Activity Theory (CHAT) as an analytical lens. CHAT derives from Soviet cultural psychology (among others Vygotsky, Leont’ev and Luria) and has been further developed (in its second and third generation) by Western scholars, including Engeström [36]. The theory stipulates that learning is collective, social, and situated in participation in practice, and that the relationship between “subjects” and “objects” is mediated by “tools”, e.g. language, physical objects and other people. Engeström expanded this unit of analysis to include three additional components, “Rules”, “Community” and “Division of labour”, and he depicted the model as an activity system - an entity of different interconnected elements which are described and illustrated in Fig. 2 [36–38]. CHAT can be used as a conceptual tool to render visible the complexity of organisations by identifying tensions and contradictions in the activities and between various factors and interacting activity systems within the organisation. The structure of the model with sub-triangles highlight the numerous relationships throughout the activity [39]. Any change in one of the components may cause changes in the others.

Through the lens of CHAT, we analysed the process of becoming a doctor, where the NGD was the “subject”, and the NGD’s aim of *fitting the white coat* was the “object”. Analysis and conceptual modelling of the NGDs’ transition into an activity system enabled us to focus on different, but interrelated aspects of the activity (system), which all had an impact on the NGDs’ process of becoming doctors. E.g. the psychological and physical tools available to the NGDs, the communities in the system (group actions), the rules and ways in which the tasks were organised. The conceptual model helped demonstrating the tensions in the activity system where NGDs are striving to fit the white coat, and it thereby pointed at possible ways to construct (and change) postgraduate medical education in a complex hospital setting. CHAT has previously proved helpful in exploring postgraduate medical education in a complex hospital setting [16, 40].

## Results and analysis

### Struggles experienced by the NGDs

The first round of analysis showed that the NGDs experienced their first months as an important learning experience, yet also stressful and challenging. As one of the participants expressed “it’s pure survival”. We were able to point out four struggles: 1) *Responsibility*; 2) *Local knowhow;* 3) *Time management* and 4) *Collaborators*. These results are shown in Table 1 and are unfolded in the following sections. The components of each of the four struggles are explored in detail (first round of analysis) and for each struggle, the related contextual factors are described (second round of analysis). Thus, we both present results from the first and second part of the analysis in the same section in order to link the contextual factors directly to the struggles and thereby showing the connection between them.

**Table 1:** Struggles experienced by the NGDs and contextual factors

Struggles	Newly Graduated Doctors’ experiences (Observed and expressed)	Contextual factors (Conceptualised by components of CHAT)
**1. Responsibility**	Overwhelmed by the sudden feeling of responsibility Fearful of (potential) consequences Difficulties and uncertainty in decision-making	Worsened when the NGDs worked physically remote from other doctors (division of labour) The NGDs are by law not the ones responsible for the final decisions (rules)
**2. Local knowhow**	Local knowhow as a prerequisite for the NGDs’ work Insufficient local knowhow affected the NGDs’ pace of work	The introduction period was time limited (rules), but with information overload (tools) Often there was no overlap between new and more experienced NGDs (rules)
**3. Time management**	Lacking a sense of time made prioritising tasks difficult Heavy workload generated stressful situations and missed learning opportunities (reflections) Many interruptions	The NGDs often covered several departments at the same time (division of labour) Guidelines caused numerous interruptions (rules)
**4. Collaborators**	Collaborators were crucial during the first months and were addressed differently Collaborators could be challenging	The NGDs had many different departments and collaborators with various perspectives to relate to (division of labour) The NGDs worked in the frontline, physically remote from their departments (division of labour)

#### Struggle 1 - responsibility

The first struggle describes how the sudden feeling of responsibility *overwhelmed* the NGDs*.* The feeling of being the ones responsible made them *fearful of (potential) consequences* and they experienced *difficulties and uncertainty in decision-making*.

##### Overwhelmed by the sudden feeling of responsibility

In general, the NGDs reported that the most evident difference between being a medical student and working as a doctor was that they were now the ones making clinical decisions and therefore feeling responsible for the patients. This was described as an overwhelming and challenging experience:

NGD10: Well, THE RESPONSIBILITY, that’s it! When you’re observing someone doing it, then you don’t learn how to do it or figure it out. It’s not something you learn by simply observing (Group interview).

NGD8: However, I do believe the first shock came on my first day. I completely shut down. I couldn’t grasp the concept of having the responsibility. [ … ] For me it was truly brutal coming from studying and then to real life. And the first shift I had … just to carry the phone (stretches out her shaking hands), I was just like that (NGD10 giggles), I was really shaking and nervous and then it goes off, and it’s a potential meningitis, and I need to head to the A&E, I don’t even know how to find it! … and then I call my attending and say: “it’s a potential meningitis”. “Well then you need to do a lumbar puncture”. And I had seen it once before, it didn’t go well, and then I had to be there by myself (NGD9 growls: hmmm) Well I was so nervous, and then the world collapsed, because the patients just kept piling in and that … I ended with completely breaking down and crying in the A&E (Group interview).

In the quote, it is clear that on her first shift on call NGD8 is assigned a patient she does not feel capable of handling, and the sudden responsibility made her very uncomfortable. Here, her breakdown was caused by the fact that she had to both attend to a potentially critically ill patient and was simultaneously required to respond to other patients.

##### Fearful of (potential) consequences

The feeling of being responsible for patients’ lives made mistakes tangible, and the awareness of matters of life and death affected the NGDs. One of the NGDs expressed how this feeling was further enhanced as medical school had taught them what the consequences of making mistakes could be. This had made him “extra alert” and wanting “everything to be done correctly”. This was also evident among other NGDs:

13.40. Finally, lunch! When seated, NGD12 is staring straight ahead. I ask her: “What’s up?”, and she tells me that she wants to confer the patient one last time with the senior doctor before she discharges him. We leave our untouched lunch in the break room and find the senior doctor in the corridor (Field note).

In this example, NGD12 had actually discharged the patient already – and conferred him with the senior doctor. Still, she felt uncomfortable with the decision and therefore turned to the senior doctor again, just to be “absolutely sure”.

##### Difficulties and uncertainty in clinical decision-making

When the participants were asked to elaborate on the differences between being a student and a junior doctor, most often the answers were that their awareness of their responsibilities made clinical decision-making difficult. Even though the NGDs all have had clerkships as students, it was still a completely different situation to be working as doctors, since as students they often just followed the doctors around and did not have the responsibility and independent interaction with patients themselves. In supplement, the NGDs discussed “having the courage [to do something]” which indicates that the fear of responsibility in decision-making is something to be overcome.

NGD14: You can easily make one … write an admission record, but you can bloody well not make a plan. I mean make decisions, you cannot figure out how to make a treatment plan when you are newly graduated as a doctor, well [ … ] I think the most difficult is to make the decisions. Well, I can see okay “I have a patient with low potassium” for example, then I must decide if the patient should get potassium. I can very well figure out that the patient needs it, but I simply cannot [ … ] anyway, I personally have difficulties making the decision if the patient should get it (Group interview).

The NGDs have read about the cases in textbooks during their studies, and they know (in theory) what to do. However, they found a barrier in making and executing the decisions themselves. This was also conspicuous in the fieldwork where the nurses would comment on all the extra scans or blood tests ordered by the NGDs, or the crowds of NGDs surrounding the senior doctor in order to ask clarifying questions.

##### Contextual factors in relation to struggle 1 - responsibility (THEORY- GUIDED)

Using the theory of CHAT, we found that the “divisions of labour” (Table 1 and Fig. 2) in the activity system influenced the NGDs’ feeling of responsibility. This was especially evident for the NGDs working across departments as *they were often physically remote from other doctors in their primary medical department.* An important part of the NGDs’ job in the A&E was to function as gatekeepers to the rest of the hospital, and the A&E is physically separated from the medical departments. The more or less permanent placement in the A&E made an NGD describe her affiliation to her own department as being “guest of the week”. This feeling was further aggravated at night, as the on call work was organised in a way where medical NGDs were the only doctors at work in the department, after just few days of training. Even though more experienced doctors were on call from home throughout the night, the NGDs still felt overwhelmed and uncomfortable by being the only doctor in the ward.

The concept of “Rules” (Table 1 and Fig. 2) was essential to bring into play when we explored the NGDs’ fear of potential consequences. By law, *the NGDs are not the ones responsible for the final decisions (regarding patients’ treatment plans)*. As long as the doctors have not yet received their authorisation to work independently as medical doctors, they work under another doctor’s responsibility [26]. This subject became evident during the observations where an NGD was requested to dictate the name of the senior doctor who she conferred the patient with, “just in case something happens”. However, this explicit guideline does not mitigate the NGDs’ overwhelming feelings:

NGD8: I’m the one who must live with it. They [senior doctors] might say, that it’s their responsibility, but I’m still the one dealing with a human life (NGD10: mmm). And that’s the thing, which to me is extremely anxiety-provoking. I don’t give a damn if the senior doctor says it’s okay or not … (Group interview).

Even though the NGDs know that they are not the ones ultimately responsible, it offers little comfort, for example when being alone and terrified at night with only a few weeks of experience, expected to prioritise between patients. In a legal sense, the NGDs might not hold the responsibility in these situations, but they still must live with the potential mistakes.

#### Struggle 2 - local knowhow

The second theme describes how the NGDs were *struggling with local knowhow as a prerequisite for their work.* At the same time, this *insufficient local knowhow affected the NGDs’ pace of work* as everything takes extra time*.*

##### Local knowhow as a prerequisite for the NGDs’ work

In the fieldwork, it became clear that local knowhow was essential in the transition from medical school to clinical practice as it was a prerequisite for working as medical doctors. For example, the NGDs had a hard time figuring out the computer system, the pager, ordering blood tests, even navigating at the hospital was a challenge:

While running [to a cardiac arrest], NGD11 says, she has no idea where the department is (Field note).

The field note was from NGD11’s first shift, and illustrates how the NGDs perform tasks they do not feel ready for and/or properly introduced to; such as holding one of the pagers for cardiac arrest when they have still not gotten to know “the house”. The NGDs often expressed frustrations about the lack of knowledge and how this insufficiency affected their work:

NGD10: I don’t think the medical skills have much to say. I think it’s ALL ABOUT local procedures, well … It’s really not much … you can almost do without knowing medical stuff, because that part you can always just call someone and ask for (NGD8: yes). You can’t call anyone and ask how to do the x-ray referral (Group interview).

NGD21: The logistics of working in a new house, that … that I think, takes up much more energy than being professional and seeing an ill patient (Group interview).

The participants experienced the local knowhow as the foundation for fitting the white coat. Only after acquiring sufficient expertise in local procedures, did they feel that their medical competencies could be put to work.

##### Insufficient knowhow affects the NGDs’ pace of work

The lack of local knowhow not only challenged the NGDs in relation to their medical expertise, it also affected the doctors’ pace of work. During the fieldwork, we observed how the more experienced NGDs worked significantly faster that the newly graduated ones. They examined the patients faster (i.e. asked quickly, precisely and without hesitation, and only examined what seemed relevant to the problem at hand), knew how to order medicine and scans, and mastered to a greater extent something as common as dictating. Both the physical surroundings and the work procedures were all unknown to the NGDs. This obviously made them work more slowly – they needed either to ask for extra help or to take the time to investigate it themselves. This caught the NGDs in a vicious circle: Everything took additional time, and this meant more waiting time, (extra) long lines of patients waiting and impatient collaborators, which all together stressed the NGDs.

##### Contextual factors in relation to struggle 2 - local knowhow (THEORY-GUIDED)

Various elements within both “rules” and “tools” (Table 1 and Fig. 2) were evident as important contextual factors. Firstly, *the introduction period of the NGDs was limited* to one or 2 weeks (“rules”), and in this limited period of time much information (“tools”) was given:

NGD11: [ … ] because it’s especially within the first week, you have to learn ALL these things, and you get SO many impressions that even though you do your best, you cannot remember anything at all (Group interview).

As the quote illustrates, the introduction period creates a paradox: On the one hand, the period is limited and fleeting; on the other hand, many of the NGDs suffer “information overload” and are unable to retain important information concerning local procedures (Table 1 and Fig. 2). This illustrates a division between being told how to do it and actually knowing how to do it; e.g. ordering a CT scan. Previously, we described how NGD8 had a breakdown in the A&E on her first shift. In the interview, the NGDs discussed how NGD8’s situation probably was accentuated by the lack of local knowhow (i.e. whom to call and when). During her first shift, she does not know (or remember) the guidelines concerning senior doctors present in the A&E. It takes time to learn to conduct oneself, and it appears difficult or downright impossible to take in the enormous amount of new information and local knowhow within the limited time of the introduction week.

Secondly, there was often *no overlap between new and more experienced NGDs* at some of the departments (“rules”, Fig. 2). When only few NGDs were employed simultaneously in the same department, and these began at the same time, it had a crucial twofold negative impact on the NGDs concerning local knowhow; both on their capabilities to acquire it and their frustrations of lacking it. The absence of overlap meant that the NGDs had no department specific experienced peer(s), and thereby there were often no or limited formal exchanges of experiences between new and more experienced NGDs. This also had the consequence that the NGDs only had a few days of formal introduction to their work before taking over the tasks themselves. The NGDs made inquiries about the opportunity for shadowing another NGD before taking over the tasks themselves, but as they were needed in the shift among colleagues, the opportunities for this were limited. Moreover, it was apparent that the new NGDs missed having a “near” peer during their first months. The informants described the peer relationship as a safe haven, which allowed asking “stupid” questions and getting mental support. NGDs employed at departments with numerous NGDs emphasised the opportunity for sparring with peers as essential in learning how to conduct oneself as doctor.

#### Struggle 3 - time management

The third theme describes how the feeling of shortage of time was yet another crucial factor for the NGDs. During their first months, they reported having an insufficient sense of time, which made *prioritising between tasks difficult*. Their work was characterised by a *heavy workload, which generated stressful situations and missed learning opportunities,* and this was furthermore exacerbated by *many interruptions*.

##### Lacking a sense of time made prioritising between tasks difficult

During the first months, the NGDs struggled with time management, e.g. about how long they spent on a task and what was a reasonable amount of time with respect to patients and collaborators:

NGD10: I just think that the sense of time is really bad in the beginning. You have no idea, how long a certain kind of patient can wait, and how much time has passed, while I’ve been standing here sweating. Well, those two things you don’t have any clue about [ … ] You don’t know either, well, how long can you let the pager beep, because it goes off constantly, while I look after whoever is critically ill right now. When you don’t know what’s realistic to do, and what the time frame is. If you don’t know that you cannot prioritise (Group interview).

In NGD10’s statement, there are two elements relating to time management. Firstly, he stated how he experienced a lack of sense of time; everything is new and “time flies”. Secondly, he had not yet learned how long the various tasks are supposed to take, and how long it is safe for the patients to wait. This makes it hard for NGDs to prioritise; e.g. between tasks and patients. At medical school, the students were taught about diseases and treatments, but the NGDs expressed how the teaching seldom included any aspects of time. This makes it hard to estimate the waiting time when colleagues from different departments call and ask them to see a patient, and this led to confrontations.

##### Heavy workload generated stressful situations and missed learning opportunities

When we asked our participants about their experiences of their first months, they stated that the heavy workload caused many stressful situations. As in most healthcare systems, the NGDs’ were often busy:

NGD6: [ … ] I don’t think I often had time to think about; what can this be, I need to look it up, how is it with this thing, what are you supposed to do? It just became; I went out and presented the patient I had, and then I needed an answer. Because I needed to move the patient to the ward … You did learn something, but probably not as much as I had thought (Group interview).

The NGDs emphasised that the lack of time had an impact on how they worked and especially when they asked for help. This was particularly apparent when there was a seemingly endless line of patients waiting, and telephones that would never stop ringing. This made the NGDs call for help more quickly and thereby choose the “easier” solution. They did not feel they had enough time to investigate symptoms, diseases etc. themselves before asking for help. One problem with this strategy was that they felt they bypassed any intermediate thought processes and thereby potentially lost time to reflect and learn from the situation. Instead of doing all the reflections and investigations themselves, they sought concrete answers from more experienced doctors and nurses to get the patients through faster.

##### Many interruptions

The heavy workload was further aggravated by the many interruptions. This is exemplified by these field notes:

10 pm: NGD17 approaches the patient, who is on the bed in a dark room because of a headache. 10.05 pm: NGD17 is paged, she walks outside, calls [ … ] 10.12 pm: Paged again. 10.16 pm: Paged again. 10.25 pm: The examination is completed (Field notes)

In this case, NGD17 was interrupted in her interaction with the patient every time the phone rang. The first two times, NGD17 left the room. Once returned, it took considerable time to resume the examination, and thus the interruptions influenced her productivity. To add injury to insult every disturbing phone call was regarding other patients – either new ones or patients waiting. NGD17 wrote all the new information (from each call) on a pad of paper in her white coat, adding new tasks to the ever-expanding to-do-list.

##### Contextual factors in relation to struggle 3 - time management (THEORY-GUIDED)

Elements within the “division of labour” and “rules” (Table 1 and Fig. 2) reinforced the difficulty with “time management”. Round the clock, the NGDs from different departments took turn in attending to all acutely ill medical patients admitted to the hospital. Some of the NGDs also had to take care of their respective departments by looking after the patients already admitted. On top of this, these NGDs were part of a working collaboration across several departments that extended through evenings, nights and weekends. Thus, they *covered several departments at the same time* (“division of labour”, Fig. 2), which entailed that during nightly hours, there was not always a doctor present in each department; the doctor might be in the A&E or another department. Thus, there were often several patients waiting in different departments, and this required the NGDs to decide which patients were most critical. This “division of labour” generated repeated calls from impatiently waiting nurses with little knowledge of when the doctor may return. These frequent disturbances cause both stress and interruption of their work as they have to respond to a multitude of issues simultaneously. To complicate this further, the medical departments and the A&E were located at opposite ends of the hospital, and consequently the NGDs must walk across the hospital premises multiple times each shift and work in various sections of the hospital. This recurrent travelling takes time – time that is not spent bedside or doing patient related work. Furthermore, the NGDs’ affiliation to different departments had an influence on their “tools”. Each department had its own pager and telephone, and thus the white coats where often filled with multiple phones/pagers.

In the exploration of the many disturbances of the NGDs, the concept of “rules” (Fig. 2) was relevant. As the hospital employs a guideline or an *early warning system* to prevent deterioration of patients’ conditions, the nurses are required (if triggered by the algorithm) to inform the doctors about various physiological parameters (e.g. temperature, oxygen saturation, blood pressure, pulse):

NGD9: Sometimes, I’m called only to be informed “the patient’s temperature is 38,6”, “okay, what was it before?”, “38,3”, “okay, anything else?”, “no, the patient is completely unaffected” [ … ]. Try to imagine a shift where you are contacted because of such minor details throughout the night (Group interview).

In these situations, the NGDs experienced being called (i.e. disturbed) with trivialities which they were required to respond to. They described how they, as newly graduated, found it difficult to act over the phone and needed to see the patient first-hand. This called for additional mileage.

#### Struggle 4 - the collaborators

The fourth and final theme describes the collaboration between the NGDs and their colleagues (both doctors and nurses). On the one hand, *the collaborators were crucial* when the NGDs struggled during their first months. On the other hand, the same *collaborators could be challenging,* especially when conflicting agendas were present.

##### The collaborators were crucial and were addressed differently

The NGDs were highly dependent on their collaborators in the process of fitting the white coat. As mentioned previously, local knowhow was an essential prerequisite for functioning in clinical practice, and since the collaborators hold this key, they become crucial in the transition.

He wants to page him [the physician on call], but on the list with all the numbers, there are only three digits listed for each person, and he is quite certain one must press five digits. “Why doesn’t it say here?”. He finds the nurse from earlier and asks him “a stupid question” (Field note).

This example shows how the NGDs were struggling and how they overcame the challenges; by turning to their collaborators often with simple, yet necessary procedures. The collaborators were peer NGDs, senior doctors and nurses. However, they engaged in three very different types of collaborations. As mentioned previously the NGDs described the peer relationship as a safe haven, where the NGDs supported each other:

NGD12: I think, it has made a difference that we are so many newly graduated doctors in the A&E (others: mmm [In acknowledgement]). You are a part of some sort of community, where there’s always someone to ask (Group interview).

NGD9: I honestly don’t know what I would have done without you guys [nearest peers] (Group interview).

It is noteworthy that the participants felt that the community with other NGDs provided safety. Because of the sense of community, they did not feel alone with the struggles they faced. For instance, it was safe to ask “stupid” questions, they would help each other with the patients, tend to each other’s wellbeing and share their feelings of insufficiency. One of the NGDs even relieved a peer by taking over the person’s pagers, even though he was not supposed or obliged to.

However, the peers were not the only ones consulted when there were struggles. The nurses were often the ones present, and since they had experience and local knowhow, the NGDs would ask the nurses about local procedures. They would consult the senior doctors when needing help in decision-making and concerning medical issues:

The interviewer: [Who do] you primarily consult during the day [ … ]?

NGD14: It all depends on the situation … If you’re having frustrations or have had an unusual experience with a patient, then it’s one of the junior doctors. However, if it’s concerning a medical issue, then it’s one of the senior [doctors] (Group interview)

This strategic selection of colleagues illustrates that when the NGDs were struggling they did not just call a random colleague, but chose their collaborators depending on the struggle at hand.

##### The collaborators could be challenging

The same collaborators that the NGDs asked for help could also be a challenge; for example, concerning patient flow and pace of work. This was especially evident with some of the nurses in the A&E:

NGD9: In addition, there is a general pressure from the A&E concerning the fact that their [the nurses] primary task is to ensure that the patients are quickly examined and shipped down the line, so the newly arrived can be seen. Additionally, there is also a time pressure from the staff; “why aren’t you here [in the A&E] yet?”, “why hasn’t the patient been transferred [to the ward] yet?”. Because in the moment the patients leave the A&E, their work [the nurses] with the patient is done, they can move on to the next. Thus, we always need to … I think, you are always made aware of “hurry up”, “get this done” (Group interview).

NGD17: I see it as two different agendas. They [the nurses] just want to empty the bed, the room and the A&E so it’s ready for the next patient, and we would like to give the patient the best treatment, [ … ] So I think, it is because they have to move on, and we would like to … we are most comfortable if we have the grand overview (Group interview).

In these examples, the NGDs experienced that the nurses in the A&E often had a different agenda from their own. The nurses wanted the patients to be, as fast as possible, ready for either discharge or admittance to another department. As described previously, the NGDs found these decisions hard to make as they felt they were the ones responsible at the end of the day. The many patients waiting and different agendas sometimes generated a tense atmosphere. Sneering was also seen at times during the observations:

When we leave the patient, NGD1 wants to find the senior doctor again. When she asks for him, one of the nurses answers, “no, there is no “grown-up” doctors here”. NGD1, slightly laughing “Grown-up doctor?”, “Yes, grown-up doctors, you know … ” the nurse answers and walks away (Field notes).

In these situations, the NGDs did not act on the harsh comment, but in the interviews, they expressed how these situations made them feel excluded or unwelcome.

##### Contextual factors in relation to struggle 4 - collaborators (THEORY-GUIDED)

The organisation where the NGDs have *many different departments and collaborators with various perspectives to relate to* (“division of labour”, Table 1 and Fig. 2) links closely to the NGDs’ experience of their collaborators being crucial. When admitting patients for further diagnosing and treatment, the NGDs encountered most of the departments in the hospital. All of which had their own demands, expectations and agendas about how the NGDs should complete the task of being gatekeepers to the hospital. The various procedures and rules made the NGDs dependent on the help from others, and at the same time put the NGDs in conflict between different departments’ guidelines:

NGD18: [ … ] And when it’s your senior doctors [employed in the A&E], there‘s one rule, and when it’s the other doctors there are other rules, I think. I don’t know if you have experienced this as well, but it’s EXTREMELY confusing (Group interview).

In this case, NGD18 perceived conflict between different departments “rules”, and thus she had to navigate these depending on which department was represented. The NGDs’ affiliation with many different departments generated many telephone calls from across the organisation, which were often about new or waiting patients. Therefore, various “Tools” (Fig. 2) become significant:

NGD17: THAT pager … It almost wakes up the entire ward [other NGDs laughing] … It doesn’t really have any inhibitions, right? It also goes off when you’re with a crying relative or in the middle of a rectal examination, and then it goes off three times while doing it, right? I really don’t like it [the pager] (Group interview).

NGD18 is very chatty on the way to the x-ray conference, and she tells me, that she has been very nervous about today. She glares down at her pager and says: “I just fear that it suddenly goes off, but it will obviously” [ … ] At the conference, NGD18 is sitting, tossing and turning her pager, and looks at it several times. Suddenly her phone rings, she is startled and goes outside to answer [ … ] “Phew”, she says, “it actually went alright” (Field note)

In the quotes, it is conspicuous that the phone and pager were not just neutral tools but were often associated with strong and sometimes negative emotions, and thus ascribed agency.

Another element within the “division of labour” (Fig. 2) was the organisation where the NGDs worked *in the frontline, physically remote from their departments* and thereby their colleagues. The NGDs described themselves as “guests” in the other departments and had the feeling of being in unknown territory. This organisation also limited the NGDs’ opportunity to make use of the benefits of working together with peers (support and talk, psychological “tool”).

## Discussion

This study explored how NGDs experience their first months of work in a complex hospital setting. The aim was to understand 1) which struggles they were facing, and 2) which contextual factors within the hospital organisation might be essential in the process of fitting the white coat.

**Fig. 3:**
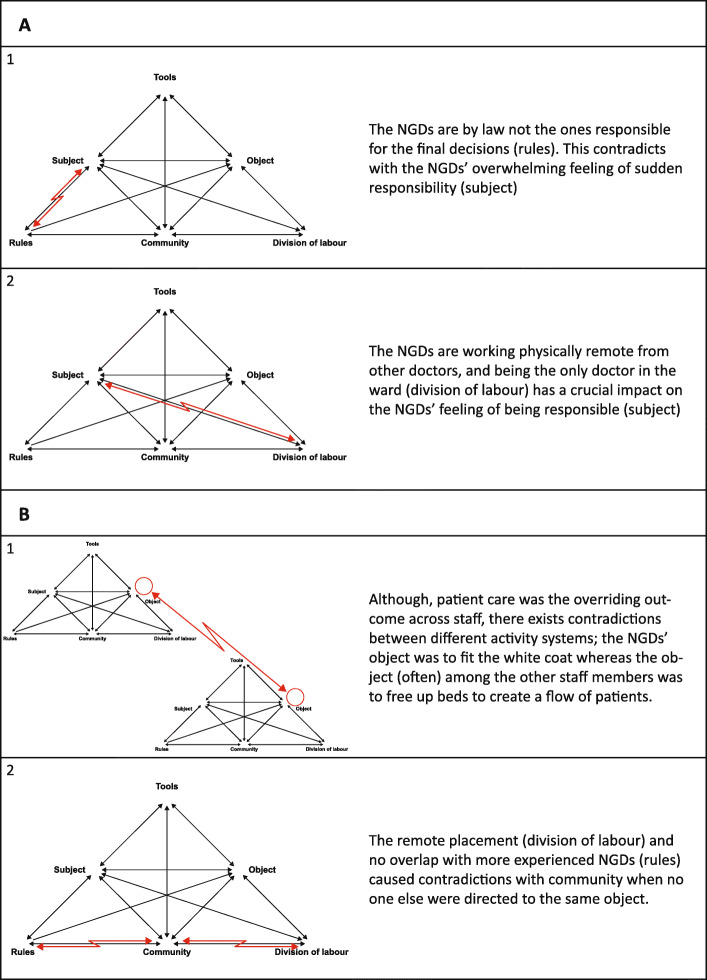
Contradictions in the activity system

The overall picture was that the NGDs experienced their first months of practice as overwhelming and completely different from their experiences as students. We showed that four struggles were of paramount importance in this transition. In the discussion, we will focus on how these are highly intertwined, interacting and influenced by one another. CHAT offers a way to discuss our findings through the concept of “contradiction”. The term “contradiction” describes tensions between different parts of the activity system or between different activity systems. Contradictions are often manifested as problems or conflicts in the activity system, but should rather be seen as opportunities for development [37]. We have chosen to discuss two topics within this concept: Responsibility and the complexity of the collaborations. In this part of the discussion, we will use the terms of CHAT (e.g. “rules” and “tools”) to clarify the contradictions within the activity system, illustrated in Fig. 3. Finally, we discuss the overall consequences that the various struggles and contextual factors had on the NGDs’ process of fitting the white coat – and possible ways to mitigate the challenges.

### Responsibility

Our results show that one of the most vital differences between being a medical student and working in the clinic as a medical doctor is the sudden feeling of responsibility. This is in line with previous research describing how the realisation that the NGDs are now responsible for the patients’ lives makes them feel burdened and fearful [8, 17, 20, 22]. As such, the theme in itself is not exceptional. The interesting thing is how this experience somehow contradicts the fact that the NGDs by law are not the ones responsible for the final decisions as they work under another doctor’s responsibility (Fig. 3.A.1). This corresponds with Kilminster et al.’s [23] description of trainees in the UK who felt responsible when left alone in the ward, despite a formal framework stating that they are actually not. During our observations the matter of responsibility was also present, e.g. when they were requested to dictate the name of the senior doctor who they conferred the patient with “just in case if something happens”. Both through the national legislation, the local guidelines and requests it is attempted to establish a framework which exonerate the NGDs from the final responsibility – but this has apparently failed. Thus, there seems to be a remaining contradiction where, regardless of the explicit legislation (“rules”), the NGDs (“subject”) still feel burdened by the responsibility of patient care. Here it is not only a matter of keeping a line of retreat open; it is dealing with the anxiety-provoking decisions in the middle of the night knowing the potential consequences for the patients. We showed how the context and the way the NGDs’ work is organised, where they are working physically remote from other doctors and being the only doctor in the ward (“division of labour”), have a crucial impact on the NGDs’ feeling of being responsible (Fig. 3.A.2).

The overwhelming feeling of responsibility is an important part in the transition, and despite many regulations, it seems to be impossible to eliminate. However, it is important to address this and organise work and learning to facilitate a community, including the one with peers, where this challenge can be addressed.

### Complexity in the collaborations

When struggling in the new role, the collaborators became the NGDs’ salvation. This corresponds with previous studies, which also describe how the pressure of the first months was eased when the junior doctors felt supported [8, 16, 22]. This includes both the peers as a safety net [5, 41], the senior doctors as the medical expertise [8, 20, 22] and the nurses as the ones with the local knowhow [8, 41].

In our study, we explored these collaborations in depth and found that it was not unequivocally an easy constellation, which has received only little attention in previous literature. Although patient care was the overriding outcome across staff, conflicting agendas and different priorities appeared when nurses wanted the patients to be ready for either discharge or admittance to another department as fast as possible, and the NGDs found these decisions hard to make. This clash created contradictions between different activity systems (Fig. 3.B.1); the NGDs’ overall aim, their “object”, was to fit the white coat whereas the “object” of the other staff members was predominately to free up beds to create a flow of patients. Since the NGDs were dependent on their collaborators, it became important for them to ensure a good relation – and this often entailed prioritising the flow of patients before their learning.

The challenges concerning the collaborators were further aggravated by the NGDs’ remote placement and thus their limited access to the community. As newcomers, the NGDs depended on support from their collaborators, but they felt left alone with no safe haven in peers or trusted senior doctors. Engeström [36] describes the component “community” as a group of individuals who all act in relation to the same object. However, the remote placement (“division of labour”) and no overlap with more experienced NGDs (“rule”) caused contradictions when no one else were directed to the same object (Fig. 3.B.2). This again strengthened their feeling of being alone and overwhelmed by the various struggles - and corresponds with an NGD’s statement of feeling as “the guest of the week”.

Our results emphasise that the members of the “community” are a pivotal part in the process of fitting the white coat, and they show how elements within “rules” and “division of labour” may limit or hinder the access to the “community”. Thus, it is important that the planning of the NGDs’ postgraduate medical education programme addresses this essential need, for example by ensuring clinical encounters between NGDs and their closest collaborators.

### The overall consequences for learning

When the *responsibility* is overwhelming, when the NGDs don’t *know how* to do things, when they are short *of time* and their *collaborators* are not available, the NGDs often chose the quickest solution; consult their collaborators for answers. This “quick fix” has implications for their opportunities to learn, as they miss out on the intermediate results and thoughts behind the decisions and skip their own important learning.

The organisation where the NGDs work full-time and at the same time are engaged in an education programme (acquiring skills) often generates a conflict between” service” and “learning”. This struggle seems to be unavoidable [1, 7, 28], however this study underlines the importance of working with various elements within the hospital organisation, which might mitigate some of the challenges.

The CHAT theory provided us with a model to identify relevant contextual factors and helped us clarifying how various elements of the activity system caused changes in the others and how the challenges this created could be addressed. This study contributes to the medical education literature by increasing our understanding of how the contextual factors influence the NGDs’ work and education environment. This knowledge is crucial to incorporate into further work of optimising the postgraduate medical education, and it may have an important implication for the undergraduate curriculum as well.

### Future perspectives

Our results suggest several contextual factors within the hospital organisation that could be addressed in order to mitigate the NGDs’ struggles when fitting the white coat. Future work is needed to explore these factors further and ideally in collaboration with all the involved stakeholders in order to contribute to new learning in the organisation and better organisation of the NGDs’ first months. By exploring the struggles and the contextual factors involved, our findings also evoked an interest in how the NGDs handled these struggles. In future studies, we aim to both explore how the NGDs “survive” and to develop appropriate initiatives to diminish some of the challenges which the NGDs are facing.

### Limitations

Our study has some limitations. Firstly, as it focused on experienced struggles, it does not pay much attention to the more positive aspects of working as an NGD. Secondly, our study was conducted in a limited number of medical specialities at a single hospital. Nevertheless, we believe that the description of this case and the referral to the various contextual factors and elements described in CHAT could allow others to recognise and address similar problems in their own institutions.

## Conclusion

In this study, we found that the NGDs experience several struggles when working as newly graduated in a complex clinical setting: *Responsibility*, *Local knowhow*, *Time management*, and *Collaborators*. We further explored various contextual factors, which might have an influence on these experiences. These findings represent a powerful demonstration of the need to take contextual factors into account when developing postgraduate medical education in order to mitigate some of the struggles that the NGDs are facing. In doing so, it is important to bear in mind that these are interrelated and when modifying one element, another may be affected.

## Data Availability

The data generated and analysed during the current study is available from the corresponding author on reasonable request.

## Abbreviations

NGD: Newly Graduated Doctor
CHAT: Cultural Historical Activity Theory
A&E: The Accident and Emergency Department
